# Dietary intake of folate, vitamin B_6_, and vitamin B_12_, genetic polymorphism of related enzymes, and risk of breast cancer: a case-control study in Brazilian women

**DOI:** 10.1186/1471-2407-9-122

**Published:** 2009-04-24

**Authors:** Enbo Ma, Motoki Iwasaki, Ishihara Junko, Gerson Shigeaki Hamada, Ines Nobuko Nishimoto, Solange Maria Torchia Carvalho, Juvenal Motola, Fábio Martins Laginha, Shoichiro Tsugane

**Affiliations:** 1 Epidemiology and Prevention Division, Research Center for Cancer Prevention and Screening, National Cancer Center, 5-1-1 Tsukiji, Chuo-ku, Tokyo 104-0045, Japan; 2 Department of Nutrition, Junior College of Tokyo University of Agriculture, 1-1-1 Sakuragaoka, Setagaya-ku, Tokyo 156-8502, Japan; 3 Nikkei Disease Prevention Center, São Paulo, Brazil; 4 Statisitical Section/Head and Neck Surgery and Otorhinolaryngology Department, Hospital AC Camargo, São Paulo, Brazil; 5 Departamento de Mastologia, Hospital AC Camargo, São Paulo, Brazil; 6 Department of Breast Surgery, Hospital Pérola Byington, São Paulo, Brazil

## Abstract

**Background:**

Several studies have determined that dietary intake of B vitamins may be associated with breast cancer risk as a result of interactions between *5,10-methylenetetrahydrofolate reductase (MTHFR) *and *methionine synthase *(*MTR*) in the one-carbon metabolism pathway. However, the association between B vitamin intake and breast cancer risk in Brazilian women in particular has not yet been investigated.

**Methods:**

A case-control study was conducted in São Paulo, Brazil, with 458 age-matched pairs of Brazilian women. Energy-adjusted intakes of folate, vitamin B_6_, and vitamin B_12 _were derived from a validated Food Frequency Questionnaire (FFQ). Genotyping was completed for *MTHFR *A1298C and C677T, and *MTR *A2756G polymorphisms. A logistical regression model was used to calculate odds ratios (ORs) and 95% confidence intervals (95% CIs).

**Results:**

Neither dietary intake of folate, vitamin B_6_, or vitamin B_12 _nor *MTHFR *polymorphisms were independently associated with breast cancer risk. Analysis stratified by menopausal status showed a significant association between placement in the highest tertile of folate intake and risk of breast cancer in premenopausal women (OR = 2.17, 95% CI: 1.23–3.83; *P*_*trend *_= 0.010). The *MTR *2756GG genotype was associated with a higher risk of breast cancer than the 2756AA genotype (OR = 1.99, 95% CI = 1.01–3.92; *P*_*trend *_= 0.801), and statistically significant interactions with regard to risk were observed between the *MTHFR *A1298C polymorphism and folate (P = 0.024) or vitamin B_6 _(P = 0.043), and between the *MTHFR *C677T polymorphism and folate (P = 0.043) or vitamin B_12 _(P = 0.022).

**Conclusion:**

*MTHFR *polymorphisms and dietary intake of folate, vitamin B_6_, and vitamin B_12 _had no overall association with breast cancer risk. However, increased risk was observed in total women with the *MTR *2756GG genotype and in premenopausal women with high folate intake. These findings, as well as significant interactions between *MTHFR *polymorphisms and B vitamins, warrant further investigation.

## Background

Folate and other methyl-related B vitamins are essential nutrients which play important roles in DNA synthesis (genetics), repair, and methylation (epigenetics). These roles in turn indicate a potential association for these vitamins with the development of several types of cancer [1-3]. For example, in one folate metabolism pathway, the enzyme *5,10-methylenetetrahydrofolate reductase (MTHFR) *irreversibly catalyzes the conversion of 5,10-methylenetetrahydrofolate (5,10-methylene THF) to 5-methyltetrahydrofolate (5-methyl THF), the methyl donor in DNA methylation. In another, folate leads to purine synthesis in DNA repair [2-4]. Activity of the *MTHFR *gene is attenuated by two common genetic polymorphisms, the *MTHFR *C677T and A1298C polymorphisms [2,5]. *Methionine synthase (MTR)*, which catalyzes the remethylation of homocysteine to methionine, has the common genetic polymorphism *MTR *A2756G [5,6].

Several studies have suggested an inverse association between folate intake and breast cancer risk in all women [7-13], pre- [7,8], and postmenopausal women [7-9,12,14,15]. However, recent meta-analysis has shown no clear overall association between folate intake or folate levels in the blood and breast cancer risk [2,16]. A number of studies have examined *MTHFR *and *MTR *polymorphisms [4-6,17-25] and found that variant genotypes of *MTHFR *C677T are associated with an increased [4,17,22-25] or decreased [5,20] risk of breast cancer, while those of *MTHFR *A1298C are associated with a decreased risk [24] and those of *MTR *A2756G with a decreased risk [6]. Several studies have further reported on the interaction between these *MTHFR *genotypes and folate intake in affecting risk [4,19]. In addition to the influence of genotype, DNA methylation and synthesis are also subject to the effects of other vitamins relevant to the one-carbon metabolism pathway, including vitamin B_6 _(pyridoxine) as a cofactor of *MTHFR *and vitamin B_12 _as a cofactor of *MTR *[4,6,19,26]. Although a role for genotype in modifying the association between dietary intake and disease is biologically plausible [2,4], research into the protective effects of increasing dietary intake of these B vitamins against breast cancer risk remains inconclusive [2,27].

In our similar previous case-control study conducted in a Japanese population, we found no significant association between dietary folate intake, *MTHFR *and *MTR *genotype, and breast cancer risk [28]. Although the incidence of breast cancer is higher in Brazilian than Japanese women [29], the association between B vitamin intake in the diet, relevant genotypes, and breast cancer risk in Brazilian populations has not been investigated. Here, we conducted a hospital-based case-control study in São Paulo, Brazil, to investigate the role of *MTHFR *and *MTR *polymorphisms in modifying the association of folate and other B vitamin intake with breast cancer risk in Brazilian women.

## Methods

### Subjects

Eligible cases were a consecutive series of female patients aged 20–74 years with newly diagnosed and histologically confirmed invasive breast cancer. Cases were recruited between 2001 and 2006 at eight hospitals in São Paulo which historically treat a relatively large number of Japanese-Brazilian patients. Eligible controls confirmed not to have any cancer were selected from the same hospitals, with one control matched to each case by age (within 5 years) and ethnicity (Caucasian, African, Asian, or mixed) during the study period. Primary reasons for study patients visiting a hospital included health check-up (52%), treatment for gynecological (13%; 64% of which were uterine myoma), urological (8%), and dermatological conditions (6%), clinical examination (8%), and others. Of the eligible cases, 472 patients (389 non-Japanese Brazilian [99%] and 83 Japanese-Brazilian [91%]) gave written informed consent, while among potential controls, 22 patients refused, resulting in a total of 472 matched pairs ultimately enrolled. The study was approved by the Comissão Nacional de Ética em Pesquisa (CONEP), Brasília, Brazil, and by the Institute Review Board of the National Cancer Center, Tokyo, Japan, in 2000.

### Data Collection and Dietary Assessment

In-person interviews were conducted by trained interviewers using a structured questionnaire which included questions on demographic characteristics, medical history, family history of cancer, anthropometric factors, smoking habits, physical activities, menstrual and reproductive factors, as well as a self-quantitative FFQ with 118 food terms.

The FFQ used here, developed specifically for this study to estimate dietary intake among participants, inquired about how often participants consumed individual food items (frequency of consumption), as well as about representative relative sizes compared with standard portions in the preceding year (control patients) or one year before their breast cancer diagnosis (cancer patients). Daily food intake was calculated by multiplying frequency by standard portion size and relative size for each food item in the FFQ. Daily intake of nutrients was calculated using the United States Department of Agriculture (USDA) food composition tables [30] and the Fifth Revised and Enlarged Edition of the Standard Tables of Food Composition in Japan [31] for intake regarding Japanese-specific foods. The regression-residual model was used to adjust intake of folate, vitamin B_6_, and vitamin B_12 _[32]. Our FFQ included questions on supplement use, but nutrient intake data from supplements were not included in analyses, as no comprehensive data for supplements were available.

The validity of nutrient intake estimated from the FFQ was evaluated in a subsample of the control group. Fifty-five women completed a 4-day dietary record (DR) in two seasons. Spearman's correlation coefficient between energy-adjusted nutrient intakes estimated from the FFQ and DR was 0.30 for folate, 0.38 for vitamin B_6_, and 0.33 for vitamin B_12_.

### Genotype of Polymorphisms

Peripheral blood samples were obtained from each subject and stored at -80°C until analysis. Genomic DNA samples were extracted from the blood using QIAGEN FlexiGene^® ^DNA kits in accordance with the manufacturer's protocol. Genotyping of the three single-nucleotide polymorphisms (SNPs) in the *MTHFR *and *MTR *genes was performed by a commercial laboratory (Genetic Lab., Inc., Sapporo, Japan) using TaqMan^® ^SNP Genotyping Assays developed by Applied Biosystems (Framingham, MA, USA). Cases and matched controls were analyzed in the same well by laboratory personnel blinded to case-control status. To assess quality control, we conducted laboratory genotyping of six SNPs of four genes (*N-acetyltransferase 2 *[*NAT2*], *cytochrome P450c17α *[*CYP17*], *aromatase *[*CYP19*], and *cytochrome P450 2E1 *[*CYP2E1*]) using approximately 25% of samples from the present study, although SNPs used in the present study were not included. The concordance rates between Genetic Lab. and our laboratory varied between 97.5% and 99.2% for the six SNPs.

### Data Analysis

Study subjects with markedly low or high total energy intake (<500 or ≥4000 kcal) or without DNA samples were excluded from the study, leaving 458 pairs of Brazilian women for analysis (379 non-Japanese Brazilian and 79 Japanese-Brazilian pairs). ORs and 95% CIs for associations between dietary intake, polymorphisms, and risk of breast cancer were calculated by the conditional logistical model, and statistical significance was examined by the Wald chi-square test. Baseline characteristics between cases and controls were compared by the Cochran-Mantel-Haenszel test using matched-pair strata. Deviation from the Hardy-Weinberg equilibrium in genotype frequencies was assessed with the chi-square test.

In the primary analysis of all subjects, risk factors identified by univariate analysis as independently related to disease risk, including smoking habit, alcohol consumption, number of live births, and a moderate level of physical activity in the preceding 5 years, were adjusted in multivariable analyses. Other potential confounders such as age at menarche, age at first live birth, breast feeding, number of pregnancies, and breast cancer in first-degree relatives were not independently associated with breast cancer risk, nor did they substantially modify the effects when tested in the multivariable model (less than 10% change in strength), and were thus not retained. Nutrient intakes of folate, vitamin B_6_, and vitamin B_12 _were categorized into tertiles for total study subjects based on the distribution among controls, with the lowest tertiles considered as the reference categories.

To increase statistical power, respective genotypes of less-frequent polymorphisms were also combined into two groups. Genotypes of *MTHFR *677CC and 1298AA, and *MTR *2756AA were considered as the referent group. With regard to evaluation of the interactive effects of SNPs and dietary intakes on breast cancer risk, the combined effects of SNPs and dietary intakes were assessed by adding the multiplicative interaction product (genotype × dietary intake) to the final model as indicator variables.

In a secondary analysis, we stratified patients by menopause status, alcohol consumption, and vitamin supplement use using an unconditional logistic model which adjusted for identified risk factors as well as age and ethnicity. Further, trend testing was conducted by treating each categorized variable as a continuous term and entering the variable into the logistic regression models. All *p *values reported were two-sided, and the significance level was set at p < 0.05. All analyses were conducted with SAS 9.02 (SAS Institute Inc., Cary, NC, USA).

## Results

Characteristics of case and control subjects at baseline are shown in Table 1. The breast cancer case group tended to include more smokers and have higher energy intake, but lower alcohol consumption, less moderate physical activity, fewer live births, and lower vitamin supplement use than the control group.

**Table 1:** Characteristics of breast cancer and control subjects at baseline in Brazilian women

	Cases (n = 458)		Controls (n = 458)		P*
	Mean ± SD^†^				
Age (matching factor), years	53.2	± 10.9	53.2	± 10.6	-
Body mass index, kg/m^2^	26.2	± 4.7	25.8	± 4.2	*0.207*
Age at menarche, years	13.1	± 1.8	13.1	± 1.8	*0.676*
Age at the first live birth, years	24.0	± 5.5	23.3	± 5.4	*0.082*
Age at menopause, years	47.8	± 6.0	47.6	± 5.6	*0.689*
					
	Number (%)				
Japanese-Brazilian ethnicity	79	(20.8)	79	(20.8)	*-*
Education to junior college or higher	112	(24.5)	111	(24.3)	*0.962*
Former and current smoker	183	(40.0)	145	(31.7)	*0.009*
Occasional and regular alcohol consumption	61	(13.3)	100	(21.8)	*0.001*
Moderate physical activity in the preceding 5 years	50	(10.9)	79	(17.3)	*0.006*
≥ 3 Live births	199	(43.5)	238	(52.0)	*0.034*
Breast cancer in first-degree relative	29	(6.3)	27	(5.9)	*0.783*
Breast feeding in all women	353	(77.1)	368	(80.4)	*0.452*
Postmenopausal women	274	(59.8)	289	(63.1)	*0.309*
Vitamin supplement use	28	(6.1)	54	(11.8)	*0.003*
					
Energy-adjusted nutrient intake	Mean ± SD^†^				
Folate, μg/day	544.8	± 160.2	529.4	± 154.6	*0.140*
Vitamin B_6_, mg/day	0.9	± 0.6	0.9	± 0.5	*0.143*
Vitamin B_12_, μg/day	7.5	± 8.7	7.0	± 5.7	*0.260*
Energy, kcal/day	1815.5	± 625.4	1722.9	± 593.6	*0.022*

^†^SD, standard deviation.

*Differences between case-control pairs in mean values were tested by the paired *t *test, and in proportions by the *Cochran-Mantel-Haenszel *test.

### B Vitamins and Breast Cancer Risk

Using the lowest tertile intake of folate, vitamin B_6_, and vitamin B_12 _as reference, no statistically significant association was observed between vitamin intake and breast cancer risk in all women (Table 2). Similar null results were found in Japanese and non-Japanese women, as well as in individuals who refrained from alcohol consumption or vitamin supplement use (data not shown). An increased risk was seen in premenopausal women in the highest tertile of folate intake (P for trend = 0.010) and in postmenopausal women in the highest tertile of vitamin B_6 _intake (P for trend = 0.047). Similarly, a statistically significant increase in risk was seen for high folate intake in premenopausal women of non-Japanese Brazilian ethnicity (in the highest tertile of folate intake, OR = 1.89, 95% CI = 1.06–3.38; P for trend = 0.037), but not in those of Japanese-Brazilian ethnicity.

**Table 2:** Association between energy-adjusted nutrient intake and breast cancer risk in Brazilian women

		Total			Premenopausal women			Postmenopausal women		
		
Nutrient intake*	Tertile	Case/Control	OR^†^	(95% CI)^†^	Case/Control	OR^‡^	(95% CI)^‡^	Case/Control	OR^‡^	(95% CI)^‡^
Folate (μg/day)	<446.2	140/152	1.00	-	43/60	1.00	-	97/92	1.00	-
	446.2-<602.2	42/153	1.05	(0.75–1.46)	58/50	1.73	(0.97–3.08)	84/103	0.77	(0.50–1.19)
	≥ 602.2	176/153	1.26	(0.89–1.78)	83/59	2.17	(1.23–3.83)	93/94	0.91	(0.59–1.41)
	*P trend*		*0.217*			*0.010*			*0.685*	
										
Vitamin B_6 _(mg/day)	<0.6	145/152	1.00	-	63/49	1.00	-	82/103	1.00	-
	0.6–<1.0	148/153	1.04	(0.74–1.46)	57/54	0.80	(0.46–1.39)	91/99	1.30	(0.85–1.98)
	≥ 1.0	165/153	1.18	(0.84–1.65)	64/66	0.81	(0.48–1.37)	101/87	1.54	(1.01–2.35)
	*P trend*		*0.384*			*0.418*			*0.047*	
										
Vitamin B_12 _(μg/day)	<3.9	165/152	1.00	-	61/49	1.00	-	104/103	1.00	-
	3.9–<7.3	143/153	0.90	(0.64–1.25)	55/56	0.77	(0.45–1.33)	88/97	0.96	(0.64–1.45)
	≥ 7.3	150/153	0.90	(0.65–1.26)	68/64	0.83	(0.49–1.41)	82/89	1.00	(0.65–1.52)
	*P trend*		*0.531*			*0.486*			*0.949*	

*Energy-adjusted intakes were categorized among controls by tertiles of folate, vitamin B_6_, and vitamin B_12 _for total Brazilian women.

^†^Adjusted for smoking status (never/ever), alcohol consumption (never/ever), moderate physical activity in the preceding 5 years (no/yes), and number of live births (nulliparous/1–2/≥3).

^‡^Adjusted for age group (20-/30-/40-/50-/60-/≥70), ethnicity (white/mixed/black/yellow), smoking status (never/ever), alcohol consumption (never/ever), moderate physical activity in the preceding 5 years (no/yes), and number of live births (nulliparous/1–2/≥3).

### Polymorphisms of *MTHFR *and *MTR *Genes and Breast Cancer Risk

Associations between polymorphisms of the *MTHFR *or *MTR *genes and breast cancer risk are shown in Table 3. No deviation from the Hardy-Weinberg equilibrium was observed in the controls. Compared to the reference group, women with the *MTR *2756GG genotype had a significantly increased risk of breast cancer (OR = 1.99, 95% CI = 1.01–3.92; P for trend = 0.801), but no other statistically significant associations were observed for all, pre-, or postmenopausal women. Similar null results were found in both non-Japanese and Japanese-Brazilian women (data not shown), although distribution of the *MTHFR *C677T polymorphism (CC, CT, and TT genotype) in control subjects differed significantly between the non-Japanese Brazilian (51%, 39%, and 10%, respectively) and Japanese-Brazilian women (35%, 52%, and 13%, respectively) (*P *= 0.038).

**Table 3:** Association between genotype of related enzymes and breast cancer risk in Brazilian women

Gene	Allele	Total			Premenopausal women			Postmenopausal women		
		
		Case/Control	OR^†^	(95% CI)^†^	Case/Control	OR^‡^	(95% CI)^‡^	Case/Control	OR^‡^	(95% CI)^‡^
MTHFR_AC	AA	269/279	1.00	-	117/107	1.00	-	152/172	1.00	-
	AC	168/157	1.11	(0.83–1.50)	63/52	1.09	(0.68–1.75)	105/105	1.11	(0.77–1.58)
	CC	21/22	1.03	(0.51–2.10)	4/10	0.39	(0.11–1.35)	17/12	1.96	(0.88–4.37)
	*P trend*		*0.543*			*0.585*			*0.161*	
	AC+CC	189/179	1.11	(0.83–1.48)	67/62	0.99	(0.63–1.55)	122/117	1.18	(0.84–1.67)
										
MTHFR_CT	CC	225/222	1.00	-	93/86	1.00	-	132/136	1.00	-
	CT	188/187	1.02	(0.77–1.36)	75/68	0.98	(0.62–1.56)	113/119	0.96	(0.67–1.38)
	TT	45/49	0.88	(0.55–1.42)	16/15	0.88	(0.40–1.95)	29/34	0.78	(0.44–1.38)
	*P trend*		*0.743*			*0.789*			*0.515*	
	CT+TT	233/236	0.99	(0.76–1.30)	91/83	0.96	(0.62–1.49)	142/153	0.92	(0.65–1.30)
										
MTR	AA	294/294	1.00	-	118/105	1.00	-	176/189	1.00	-
	AG	135/149	0.81	(0.59–1.10)	53/56	0.76	(0.47–1.23)	82/93	0.93	(0.64–1.35)
	GG	28/15	1.99	(1.01–3.92)	13/8	1.34	(0.51–3.51)	15/7	2.28	(0.88–5.90)
	*P trend*		*0.801*			*0.738*			*0.543*	
	AG+GG	163/164	0.92	(0.68–1.23)	66/64	0.83	(0.53–1.31)	97/100	1.02	(0.71–1.46)

^†^Adjusted for smoking status (never/ever), alcohol consumption (never/ever), moderate physical activity in the preceding 5 years (no/yes), and number of live births (nulliparous/1–2/≥3).

^‡^Adjusted for age group (20-/30-/40-/50-/60-/≥70), ethnicity (white/mixed/black/yellow), smoking status (never/ever), alcohol consumption (never/ever), moderate physical activity in the preceding 5 years (no/yes), and number of live births (nulliparous/1–2/≥3).

We also examined the effects of combined genotypes of *MTHFR *A1298C and C677T, *MTHFR *A1298C and *MTR *A2756G, and *MTHFR *C677T and *MTR *A2756G, none of which was statistically significantly associated with the risk of breast cancer compared to their wild genotypes.

### Gene-environment Interactions in Breast Cancer

The joint effects of *MTHFR *and *MTR *polymorphisms and dietary intake on breast cancer risk are shown in Table 4, in which the genotypes *MTHFR *1298AA and 677CC and *MTR *2756AA with the lowest tertiles of dietary intake were used as references, with variant allele combinations. Statistically significant positive associations between folate intake and breast cancer risk were observed among women with the *MTHFR *1298AA (P for trend = 0.003), 677CT and TT (P for trend = 0.011), and *MTR *2756AA genotypes (P for trend = 0.049). An increased risk of breast cancer was seen in those women with the lowest folate intake who had the *MTHFR *1298AC and CC genotype (OR = 1.95, 95% CI = 1.18–3.22). In contrast, a decreased risk was seen in those women with the lowest folate intake who had the *MTHFR *677CT and TT genotype (OR = 0.60, 95% CI = 0.37–0.97). Statistically significant interactions were detected for folate intake with *MTHFR *A1298C (P for interaction = 0.024) and C677T polymorphisms (P for interaction = 0.043).

**Table 4:** Combined effects of genotype of related enzymes and energy-adjusted nutrient intakes in all breast cancer cases and controls in Brazilian women

Gene	Allele	Tertile 1 (lower)*			Tertile 2			Tertile 3			*P*_*trend*_	*P*_*interaction*_
				
		Case/Control	OR^†^	(95%CI)^†^	Case/Control	OR	(95%CI)	Case/Control	OR	(95%CI)		
Folate												
MTHFR_AC	AA	72/105	1.00	-	87/89	1.40	(0.92–2.14)	110/85	1.76	(1.14–2.72)	*0.003*	*0.024*
	AC+CC	68/47	1.95	(1.18–3.22)	55/64	1.31	(0.79–2.17)	66/68	1.45	(0.88–2.41)	*0.109*	
												
MTHFR_CT	CC	73/60	1.00	-	62/75	0.72	(0.44–1.17)	90/87	0.84	(0.52–1.37)	*0.417*	*0.043*
	CT+TT	67/92	0.60	(0.37–0.97)	80/78	0.86	(0.53–1.40)	86/66	1.08	(0.66–1.77)	*0.011*	
												
MTR	AA	86/101	1.00	-	95/102	1.14	(0.75–1.72)	113/91	1.56	(1.01–2.43)	*0.049*	*0.283*
	AG+GG	54/51	1.21	(0.73–2.00)	46/51	1.12	(0.66–1.90)	63/62	1.07	(0.65–1.77)	*0.574*	
												
Vitamin B_6_												
MTHFR_AC	AA	73/82	1.00	-	85/106	0.99	(0.63–1.55)	111/91	1.50	(0.95–2.37)	*0.061*	*0.043*
	CA+CC	72/70	1.28	(0.79–2.06)	63/47	1.56	(0.92–2.63)	54/62	1.02	(0.61–1.72)	*0.669*	
												
MTHFR_CT	CC	78/68	1.00	-	68/75	0.80	(0.48–1.33)	79/79	0.90	(0.55–1.48)	*0.914*	*0.273*
	CT+TT	67/84	0.71	(0.43–1.16)	80/78	0.91	(0.56–1.46)	86/74	1.03	(0.64–1.67)	*0.105*	
												
MTR	AA	91/95	1.00	-	98/100	1.04	(0.67–1.61)	105/99	1.18	(0.77–1.81)	*0.435*	*1.000*
	AG+GG	54/57	0.92	(0.57–1.50)	50/53	0.95	(0.57–1.59)	59/54	1.07	(0.64–1.79)	*0.538*	
												
Vitamin B_12_												
MTHFR_AC	AA	96/94	1.00	-	76/96	0.78	(0.50–1.20)	97/89	1.10	(0.72–1.68)	*0.662*	*0.088*
	CA+CC	69/58	1.23	(0.76–2.00)	67/57	1.22	(0.77–1.96)	53/64	0.79	(0.49–1.30)	*0.167*	
												
MTHFR_CT	CC	83/70	1.00	-	79/70	0.90	(0.55–1.47)	63/82	0.59	(0.36–0.97)	*0.073*	*0.022*
	CT+TT	82/82	0.76	(0.46–1.24)	64/83	0.62	(0.37–1.05)	87/71	1.00	(0.63–1.60)	*0.349*	
												
MTR	AA	105/94	1.00	-	94/105	0.79	(0.52–1.20)	95/95	0.90	(0.59–1.38)	*0.560*	*0.592*
	AG+GG	60/58	0.81	(0.51–1.31)	48/48	0.92	(0.54–1.57)	55/58	0.76	(0.47–1.23)	*0.923*	

*Energy-adjusted intakes were categorized among controls by tertiles of folate, vitamin B_6_, and vitamin B_12_, respectively.

^†^Adjusted for smoking status (never/ever), alcohol consumption (never/ever), moderate physical activity in the preceding 5 years (no/yes), and number of live births (nulliparous/1–2/≥3).

A marginally positive association was observed between the highest tertile of vitamin B_6 _intake and breast cancer risk among women with the *MTHFR *1298AA genotype (P for trend = 0.061), whereas an inverse association was observed between the highest tertile of vitamin B_12 _intake and breast cancer risk among those with the *MTHFR *677CC genotype (P for trend = 0.073). A statistically significant interaction was detected between the *MTHFR *A1298C polymorphism and vitamin B_6 _intake (P for interaction = 0.043) and between the *MTHFR *C677T polymorphism and vitamin B_12 _intake (P for interaction = 0.022).

## Discussion

In this study, we found no overall association between folate, vitamin B_6, _and vitamin B_12 _intake and the risk of breast cancer. These findings are consistent with previous studies on folate [4,14,16,20,28,33-40], vitamin B_6 _[4,12,28,35,39,40], and vitamin B_12 _[8,15,28,39,40].

Generally, folate is able to prevent the development of tumors before preneoplastic lesions have been established, but conversely increases tumorigenesis once lesions have been established [39,41,42]. Although increased folate intake may be beneficial in populations deficient in this nutrient, increased intake in women with already-sufficient levels of folate may provide no further benefit, or actually be harmful [34]. Folate intake in Brazilian women in the present study (inter-tertile range, 446.2–602.2 μg/day) was higher than that measured in a study of Japanese women (inter-tertile range, 386–501 μg/day) [28] and in a meta-analysis of Caucasian and Asian women (inter-tertile range, 77–255 to 147–359 μg/day and 132–304 to 206–473 μg/day) [2]. In this study, we observed an increased risk of breast cancer in premenopausal women with high folate intake. Folate levels may have been already sufficient in the lower intake group, and thus an increase in intake may on this basis have contributed to tumorigenesis.

Similarly, a study on plasma folate found that concentration was associated with an increase in breast cancer risk in premenopausal but not in postmenopausal women [39]. This study also indicated that the proliferation rate of breast epithelial cells may be higher in premenopausal than postmenopausal women, leaving less time for DNA repair in premenopausal women [39]. Further, a large population-based case-control study reported that high dietary vitamin B_6 _intake was associated with an increased risk of breast cancer, but did not indicate the menopausal status of participants [8]. In contrast, another study found a decreased risk of breast cancer in postmenopausal women with high plasma levels of vitamin B_6 _[39]. Our finding of a significant increase in breast cancer risk in postmenopausal women due to vitamin B_6 _intake may thus be due to chance.

A Canadian study found that a high total folate intake, obtained mainly from nutritional supplements, significantly increased breast cancer risk by 32% (highest >853 μg/day versus lowest quintile ≤336 μg/day) in postmenopausal women [33]. The authors hypothesized that high folic acid concentrations may contribute to epigenetic changes in gene-regulatory mechanisms, which may result in gene silencing and enhanced cancer development, or may promote the growth of tumors expressing folate receptors [33,41]. However, the mechanism of this effect remains unclear [41]. In our study, however, overall vitamin supplement users (defined as more than once-weekly use for at least one year) accounted for 11.8% of controls, and the distribution of supplement users was similar in the highest and lowest intake groups (data not shown). Because folate and other B vitamin intake in our population was thus obtained primarily from unfortified diets, the increased risk of breast cancer in premenopausal women with high folate intake was unlikely due to supplement use.

A recent meta-analysis indicated no overall significant association between the *MTHFR *C677T polymorphism and breast cancer risk in Caucasians and East Asians, or between the *MTHFR *A1298C polymorphism and risk in Caucasians [27]. Our study supports the lack of association found in this meta-analysis as well as in others studies in Caucasian and Asian women [1,5,43]. In contrast to another finding of the meta-analysis [27], however, which found an increased risk of breast cancer in premenopausal women with the *MTHFR *677CT and TT genotype compared to those with the CC genotype, we did not observe any increase in risk in these groups. Further, contrary to the findings in a previous study conducted in Americans [1], we did not find an increased risk of breast cancer to be related to the genotype combination of *MTHFR *A1298C and C677T. This lack of concordance in findings may be due to the low proportion of the *MTHFR *677T genotype in our study population or to compensation by the folate-replete status of patients for the effects of the variant alleles of the *MTHFR *A1298C and C677T genotypes [2]. Nevertheless, few studies have achieved conclusive results regarding whether functional polymorphisms in one-carbon metabolizing genes affect breast cancer risk associated with dietary intake of folate and other related B vitamins, the biological plausibility of this scenario notwithstanding [2].

The *MTR *gene contributes to alterations in the plasma levels of homocysteine and folate; specifically, subjects with the 2756AG and GG genotypes have lower levels of plasma homocysteine or higher levels of serum folate than those with the 2756AA genotype [44,45]. When methionine levels are adequate, these variant polymorphisms with lower enzyme activity may also result in elevated homocysteine levels and DNA hypomethylation [46]. A Polish study reported the possible association between the hetero- and homozygote variants of the *MTR *A2756G and a decreased risk of breast cancer [6]. In the present study, however, we observed an increased risk of breast cancer in total subjects who had the MTR 2756GG genotype, a finding similar to that reported in a recent study conducted in Taiwan [46]. This observed increase may be due to the linkage between the *MTR *A2756G polymorphism and other genetic polymorphisms, or to the interaction between the *MTR *A2756G polymorphism and other genetic polymorphisms related to folate metabolism, such as that for methionine synthase reductase (*MTRR *G66A) [25,45], cytosolic serine hydroxymethyltransferase (*cSHMT *C1420T) [21], or *MTHFR *C677T [47]. However, we observed no statistically significant associations between the combined genotypes of *MTR *A2756G and either *MTHFR *A1298C or C667T and breast cancer risk, nor could we test the joint effects of *MTR *with *MTRR *or *cSHMT *genes since *MTRR *and *cSHMT *genes were not available in the present study. Given the low frequency of the *MTR *2756GG genotype in our study population, the significant finding of our present study may be the result of chance.

Previous authors have speculated that interactions between folate and *MTHFR *polymorphism may be elicited to show that (a) the lower activity of variant genotypes may increase the risk of breast cancer at low levels of dietary folate since less 5-methyl THF is made available for DNA methylation, and (b) variant genotypes might provide advantages over the wild genotype in folate-replete conditions since 5,10-methyl THF would be available for nucleotide synthesis [2,4]. Supporting notion (a), we found an increased risk of breast cancer among women with the lowest folate intake who had the *MTHFR *1298AC and CC genotype. Contrary to (a), however, we also saw a decrease in breast cancer risk among women with the lowest folate intake who had the *MTHFR *677CT and TT genotype. Similar findings of decreased breast cancer risk in variant *MTHFR *C677T genotypes have been reported in previous studies in German [5], Japanese-American [20], and Chinese patients [10]. The reduced activity of the *MTHFR *C677T polymorphism was speculated to increase the pool of intracellular 5,10-methylene THF, which protects against potential defects in DNA synthesis, although circulating folate levels (5-methyl THF) tended to be lower [10]. Nevertheless, the inconsistencies in results between this and previous studies may be due to differences in ethnicity [10,19], background dietary status [19,37], or possibly chance. Given the above discussion regarding folate intake level, the increased risk of breast cancer observed here in patients with the *MTHFR *1298AA and *MTR *2756AA genotype in a higher tertile of folate intake may be due to the harmful effects brought about by excessive intake of folate. Notion (b) suggests that the lower activity of variant alleles of *MTHFR *A1298C provides advantages over the *MTHFR *1298AA genotype, but with the compensation of sufficient folate intake, the lower enzyme activity was attenuated to result in the lack of significant association seen with the variant *MTHFR *A1298C genotypes in the higher category of folate intake. In addition, the observed trend to a decline in breast cancer risk with increased vitamin B_12 _intake (P for trend = 0.073) and significant interaction between vitamin B_12 _and *MTHFR *C677T polymorphism might be due to the function of vitamin B_12 _as a cofactor in folate metabolisms [12,19].

Several limitations of this study warrant mention. First, dietary intake of folate, vitamin B_6_, and vitamin B_12 _was assessed after breast cancer diagnosis and was therefore sensitive to recall bias. Case subjects may have been more motivated to recall diet than controls, and their recall may also have been inaccurate, depending on the belief of an association between diet and their disease. Second, although substantially high participation rates among both eligible case and control subjects minimized potential selection bias, the use of cancer-free patients as controls, whose dietary habits may have differed from the general population due to health consciousness or disease concerns, might have lead to selection bias. Third, as our subjects were recruited from eight hospitals in São Paulo, Brazil, they may not necessarily be representative of the general Brazilian population, and thus any extrapolation of results to the general population should be done cautiously. Fourth, our validation study found relatively low correlation coefficients between FFQ and DR-based nutrient intake estimates. Further, the validation study indicated that measurement errors in assessing nutrient intake by FFQ were unavoidable, although such random errors tend to result in null associations [48]. Fifth, although our quality control assessment of the genotyping showed high concordance rates between Genetic Lab. and our laboratory, we did not genotype the duplicate blinded samples of SNPs used in the present study and therefore cannot rule out the possibility of misclassification in genotyping status, although its effect on the results might be minimal. Sixth, stratified analyses were performed based on a relatively small number of cases, possibly limiting the interpretability of our results.

## Conclusion

In this case-control study conducted in Brazilian women, we found no overall significant associations between dietary intake of folate, vitamin B_6_, and vitamin B_12_, *MTHFR *genotype, and breast cancer risk. However, we did identify an increased risk of breast cancer in total women with the *MTR *2756GG genotype, increased risk associated with higher folate intake in premenopausal women, and gene-nutrient interactions. Further studies into these factors will help to elucidate the etiology of breast cancer.

## Abbreviations

CI: confidence interval; FFQ: food frequency questionnaire; MTHFR: 5,10-methylenetetrahydrofolate reductase; MTR: methionine synthase; OR: odds ratio; SNPs: single-nucleotide polymorphisms.

## Competing interests

The authors declare that they have no competing interests.

## Authors' contributions

EM performed the data analysis and drafted the manuscript. MI participated in the study design, coordinated the study and helped with data integrity, analysis, interpretation, and preparation of the manuscript. JI participated in data analysis and interpretation of data. GH involved the study design, supervised the study, contributed to data acquisition and interpretation. IN involved the study design, and contributed to data acquisition and interpretation. SC contributed to data acquisition and interpretation. JM contributed to data acquisition and interpretation. FL contributed to data acquisition and interpretation. ST designed, supervised and coordinated the study and helped with data interpretation and preparation of the manuscript. All authors have read and approved the final manuscript.

## Pre-publication history

The pre-publication history for this paper can be accessed here:

http://www.biomedcentral.com/1471-2407/9/122/prepub
